# Hypothesis Testing and Power Calculations for Taxonomic-Based Human Microbiome Data

**DOI:** 10.1371/journal.pone.0052078

**Published:** 2012-12-20

**Authors:** Patricio S. La Rosa, J. Paul Brooks, Elena Deych, Edward L. Boone, David J. Edwards, Qin Wang, Erica Sodergren, George Weinstock, William D. Shannon

**Affiliations:** 1 Division of General Medical Sciences, Department of Medicine, Washington University School of Medicine, St. Louis, Missouri, United States of America; 2 Department of Statistical Sciences and Operations Research, Virginia Commonwealth University, Richmond, Virginia, United States of America; 3 The Genome Institute, Washington University School of Medicine, St. Louis, Missouri, United States of America; Utah State University, United States of America

## Abstract

This paper presents new biostatistical methods for the analysis of microbiome data based on a fully parametric approach using all the data. The Dirichlet-multinomial distribution allows the analyst to calculate power and sample sizes for experimental design, perform tests of hypotheses (e.g., compare microbiomes across groups), and to estimate parameters describing microbiome properties. The use of a fully parametric model for these data has the benefit over alternative non-parametric approaches such as bootstrapping and permutation testing, in that this model is able to retain more information contained in the data. This paper details the statistical approaches for several tests of hypothesis and power/sample size calculations, and applies them for illustration to taxonomic abundance distribution and rank abundance distribution data using HMP Jumpstart data on 24 subjects for saliva, subgingival, and supragingival samples. Software for running these analyses is available.

## Introduction

The NIH Human Microbiome Project (HMP) [1] aims at characterizing, using next generation sequencing technology, the genetic diversity of microbial populations living in and on humans, and at investigating their roles in the functioning of the human body, such as their effects in nutrition and susceptibility to disease [2]. In just a few years, much work has been done to optimize the processes for collecting microbiome samples, processing the DNA, running the sequencing technology, and generating taxonomies/phylogenies from these sequences [3]. These developments will facilitate access to microbiome technology for laboratories of all sizes, enabling application in varied fields of biology, from agriculture to human disease research. However, the biostatistical analysis of metagenomic data is still being developed. Several methods to analyze metagenomic data have been proposed based on exploratory cluster analysis, bootstrap or resampling methods, and application of univariate and non-parametric statistics to subsets of the data [4]–[12]. However, these methods require a significant reduction of information, such as Unifrac [7] which reduces sequence data to pairwise distances, or ignoring correlations and the multivariate structure inherent in microbiome data, such as Metastats [12] which does univariate ‘one-taxa-at-a-time’ analyses.

Given the multivariate nature of the metagenomic data, having multivariate analysis tools is becoming important in the microbiome research community. Microbiome researchers are interested in testing multivariate hypotheses concerning the effects of treatments or experimental factors on whole assemblages of bacterial taxa, and in estimating sample sizes for such experiments. These types of analyses are useful for studies aiming at assessing the impact of microbiota on human health and on characterizing the microbial diversity in general. Statistical methods to design and analyze such studies will contribute to the translation of microbiome research from technical (bench) development to clinical (bedside) application.

The focus of this work is to develop multivariate methods to test for differences in bacterial taxa composition between groups of metagenomic samples. Multivariate non-parametric methods based on permutation test such as Mantel test [13], [14], Analysis of Similarity (ANOSIM) [15], and NP-Manova [16] are widely used among community ecologists for this purpose. However, although these three methods are attractive when a parametric distribution of the data is unknown, we believe they are not always appropriate for analyzing microbiome data. First, although a hypothesis of group difference can be tested, the results of these tests are difficult to interpret since they cannot quantify the size of the difference between the groups in terms of bacterial taxa composition. Second, permutation tests work under the assumption that the dispersion (variability) of samples within groups is the same in all groups [16], a strong assumption which when violated can lead to inflation of type I error. Third, non-parametric methods are usually less powerful than parametric methods, so when a parametric alternative is available it should be the preferred method to model metagenomic data.

In this paper, we present biostatistical methods for the analysis of microbiome data based on a fully multivariate parametric approach. In particular, the parametric model used in this paper is the Dirichlet-Multinomial distribution which has been shown recently to model metagenomic data well. In [17] the authors apply the Dirichlet-multinomial mixture for the probabilistic modeling of microbial metagenomics data, which was used to successfully cluster communities into groups with a similar composition. However, a multivariate hypothesis testing framework to compare populations using this model was not derived. In this work, we apply a different parameterization of Dirichlet-multinomial model to the one presented in [17], which is suitable to perform hypothesis testing across groups based on difference between location (mean comparison) as well as scales (variance comparison/dispersion). Using this model, we develop methods to perform parameter estimation, multivariate hypothesis testing power and sample size calculation. An open source R statistical software package (‘HMP: Hypothesis Testing and Power Calculations for Comparing Metagenomic Samples from HMP’) for fitting these models and tests is available [18].

In addition, the methods developed here are not constrained by computational resources and work for any size microbiome dataset (e.g., number of sequence reads and samples). These methods and are also likely applicable to phylogenetic analysis which is currently being investigated.

## Materials and Methods

### Ethics Statement

Subjects involved in the study provided written informed consent for screening, enrollment and specimen collection. The protocol was reviewed and approved by the Institutional Review Board at Washington University in St. Louis. The data were analyzed without personal identifiers. Research was conducted according to the principles expressed in the Declaration of Helsinki.

### Human Microbiome Data

Human microbiome data analyzed in this paper are from the subgingival, supragingival, and saliva oral sites of 24 subjects (male and female), 18–40 years old, from two geographic regions of the US: Houston, TX and St. Louis, MO [19]. The analyses presented here illustrate how the Dirichlet-multinomial biostatistical analysis is used with real data. Approximately 1×10^5^ sequences were obtained from the V1–V3 and V3–V5 variable regions of the 16S ribosomal RNA gene, and collapsed into a single sample. The sequencing was performed at one of four genome sequencing centers (J. Craig Venter Institute, Broad Institute, Human Genome Sequencing Center at Baylor, and Genome Sequencing Center at Washington University in St. Louis). Sequence reads were assigned to bacterial taxa using the Ribosomal Database Project (RDP) classifier [20], which provides a confidence score for each taxonomic classification. Only taxa labels with a confidence score > = 80% were retained in this analysis, and taxa labels below this threshold were relabeled as unknown. Although the choice of an 80% threshold on the confidence score is arbitrary, in [21] it was shown that threshold ranging between 50% to 90% provided an average classification performance of between 77% at the genus level up to 97% at the phylum level.

### Statistical Model for HMP Data

#### Dirichlet-multinomial model

Consider a set of microbiome samples measured on 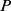 subjects with 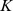 distinct taxa at an arbitrary level (e.g., phylum, class, etc.) identified across all samples. Not all taxa need to be found in all samples. Let 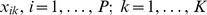 be the number of reads in subject 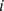 for taxon *k*, and let be the taxa count vector obtained from sample 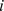. Note that 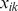 is 0 when taxon *k* is not in sample 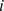. Let 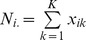 be the total number of sequence reads in sample 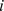, 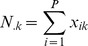 be the total number of sequence reads for taxon 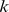 across all samples, and be the total number of sequences over all samples and taxa. Table 1 shows the format of an RDP-mapped microbiome data set.

**Table 1:** Format of a microbiome data set for 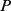 subjects and 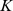 distinct taxa at an arbitrary level (e.g., Phylum, Class, etc.).

	Taxa				
Sample	1	2	…	*K*	*Total*
1	*X*_11_	*X*_12_	…	*X*_1 *K*_	*N*_1*_
2	*X*_21_	*X*_22_	…	*X*_2 *K*_	*N*_2*_
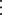	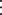	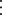	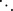	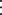	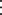
*P*	*X_P_*_1_	*X_P_*_2_	…	*X_PK_*	N_P__*_
**Total**	N_*1_	N_*2_	…	N_*K_	N_**_

Count data such as this is routinely analyzed using a multinomial distribution which is appropriate when the true frequency of each category (e.g., each taxon in microbiome data) is the same across all samples. This implies that as the number of sample points increases (i.e., number of reads) within each sample, taxa frequencies in all samples converge to the same value (e.g., all samples converge onto 40% taxa A, 25% taxa B,…) with no variability between samples. When the data exhibit overdispersion this convergence result does not occur (i.e., taxa frequencies in all samples do not converge to the same values), and the multinomial model is incorrect [22]. Hypothesis testing based on the multinomial model in the presence of overdispersion can result in an increased Type I Error (i.e., saying the microbiome samples are different when they are not) [23].

The Dirichlet-multinomial distribution prevents Type I Error inflation by taking into account the overdispersion in count data in the form displayed in Table 1. It can be characterized by the following two set of parameters [24]: 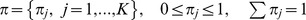 which is a vector of the expected taxa frequencies, and 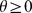 which is a number indicating the amount of overdispersion. Using this parameterization, the Dirichlet-multinomial distribution is defined as [24]:
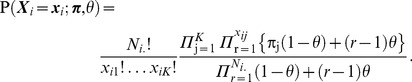
(1)


The above parameterization of the Dirichlet-multinomial distribution is suitable to perform hypothesis testing across groups based on difference between locations (comparisons of 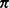 vectors) as well as scales (comparison of 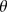 values). Other parameterizations of the Dirichlet-multinomial distribution can be found in [23], [25]. Note that the Dirichlet-multinomial distribution is a generalization of the multinomial model, which results when 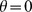. When 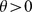 the data variability is larger than what is expected from the multinomial distribution, and the Dirichlet-multinomial distribution provides a better fit to the data.

On a side note, if the elements of the taxa count vector, 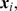 obtained from a sample are ranked (i.e., 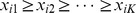), then the Dirichlet-multinomial can be used to model the rank abundance distributions (RAD) vector across samples. This is useful if the analyst is interested in comparing community structure and complexity across microbiome samples and body sites, but not interested in the names of the community members [26]–[28]. If the elements of the taxa count vector, 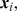 obtained from a sample are not ranked (i.e., 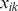 has the same taxa label across all samples), then we are modeling the abundance of species keeping their labels. This type of analysis is useful to compare community composition across microbiome samples and body sites, and it is usually referred to as analysis of species composition data [29]. Since we are interested in analyzing different taxonomic levels, we will refer to this as analysis of taxa composition data. The interested reader is referred to [26]–[29] and references therein for more details on the importance and applications of taxa composition data and RAD data analyses to study biodiversity.

#### Estimating 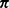 and 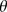


Referring to the data structure in Table 1 on a set of 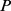 samples with counts on 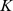 taxa, we compute the frequency of taxon 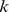 in sample 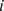 as the percentage of reads within that sample that belong to that taxa (i.e., 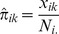). The elements of the parameter 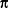 are then computed as the weighted average of the taxa frequency from each sample (i.e., 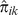) with weights given by proportion of the number of reads in sample 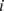 with respect to the total number of sequence reads (i.e., 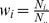).

**Figure 1:**
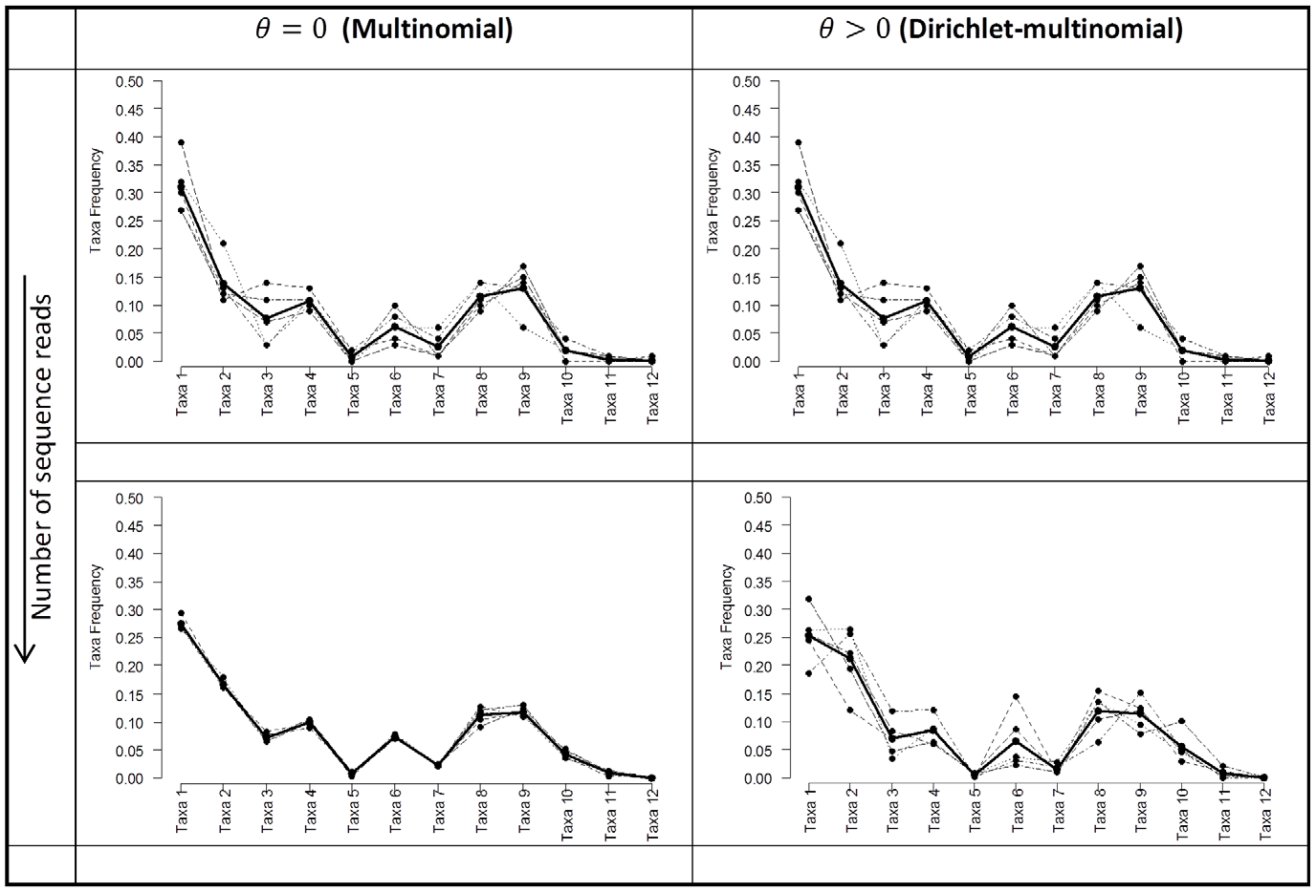
Description of Dirichlet-multinomial parameters. Intuitive description of the meaning of the overdispersion parameter 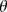. The four plots show the taxa frequencies 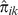 for each of the five hypothetical samples (dashed lines) with 12 taxa in each sample, and the corresponding weighted average across the five samples given by the vector of taxa frequencies 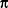 (solid line). The plots on the left show the taxa frequencies of samples drawn from a Multinomial distribution 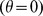 and the plots on the right show taxa frequencies of five samples drawn from a Dirichlet Multinomial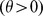. The top row of plots is for samples with a smaller number of sequence reads, while the bottom row of plots is for samples with a larger number of sequence reads. As the number of reads increases for the multinomial distribution increases each samples taxa frequencies converge onto the mean, while for the Dirichlet-multinomial an increased number of reads is still associated with the same variability between the individual samples.

To understand the overdispersion parameter 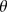 a graphical example is shown. In Figure 1 we have four plots showing the taxa frequencies 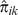 for each of the five hypothetical samples (dashed lines) with 12 taxa in each sample, and the vector of taxa frequencies 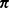 (solid line). The plots on the left correspond to taxa frequencies of five samples drawn from a multinomial distribution 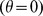 and the plots on the right correspond to taxa frequencies of five samples drawn from a Dirichlet-multinomial 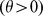. The top row of plots is for samples with a smaller number of sequence reads, while the bottom row of plots is for samples with a larger number of sequence reads. As the number of sequence reads increases the multinomial samples get closer and closer to the 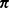, while the Dirichlet-multinomial samples continue to show variability and no convergence onto 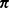. This pattern will hold true in the Dirichlet-multinomial distribution no matter how large the number of sequence reads becomes.

Given taxa counts vectors 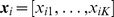 for 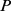 subjects, denoted in vector form as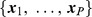 (see Table 1), the set of parameters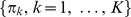 and 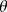 can be estimated using either the method of moments [24], [25], [30] or maximum likelihood estimation (MLE) [24] computational procedures. The method of moments estimators of 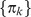 are [25]

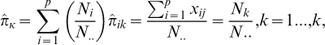
(2)


and of 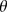 is [24], [30]

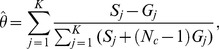
(3)where 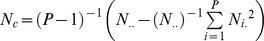, and 
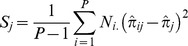
, and 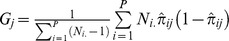 with 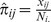. Alternatively, the MLEs 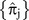 and 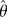 are given by 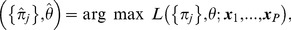
(4)where 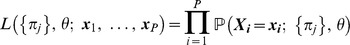 is the Dirichlet-multinomial likelihood function. The method of moments and MLE estimation procedures perform equally well in terms of statistical properties (e.g., bias, variance) for the number of subjects and reads we routinely encounter in our microbiome studies. These results are available from the authors as a Technical Report.

#### Multinomial versus Dirichlet-multinomial test

Since the presence of overdispersion increases the Type 1 Error if not controlled for, it is good to test if overdispersion is present in a set of microbiome samples. This can be done by formally testing the null hypothesis 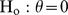 (implying no overdispersion) versus the alternative hypothesis 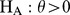 (implying overdispersion is present). An optimal test-statistic calculated from the raw metagenomic data (see Table 1) for this hypothesis is the following [31]:
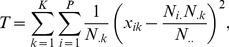
(5)which approaches a Chi-square distribution with 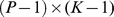 degrees of freedom when the number of sequence reads is large and the same in all samples. In the case that the number of reads varies across samples (such as in microbiomes samples) the test statistics converges to a weighted Chi-square with a modified degree of freedom (see [31] for more details). This is a more complicated formulation and is not presented here, but an approximate solution presented in [31] has been included in the R HMP Package. Note that this hypothesis test establishes that the data are better represented by a Dirichlet-multinomial than a multinomial. However, it does not affirm than Dirichlet-multinomial fits the data best. A goodness-of-fit test statistic for doing this is currently being derived.

### Hypothesis Testing

#### Comparing 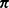 to a previously specified microbiome population

Consider the problem of comparing microbiome samples to a vector of taxa frequencies 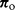 gathered in an earlier study or hypothesized by the investigator. This might be done to test if new samples come from e the same or different population from earlier samples, such as comparing a population to the HMP healthy controls. This test is analogous to a one sample t-test in classical statistics, which, in our case, corresponds to assessing whether the vector of taxa frequencies 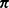 for the new samples, estimated using method of moments or MLE, are equal to the taxa frequencies vector 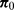 from the previously studied population.

The following statistic formally tests the hypothesis 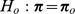 versus the alternative that 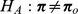: [32]

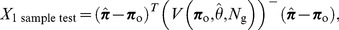
(6)which is a generalized Wald test statistic where 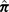 is an unbiased estimator of 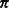, 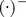 is the Moore-Penrose generalized inverse, and 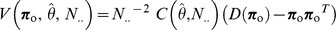 with 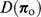 a diagonal matrix with diagonal elements given by 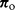 and 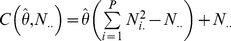, and where 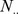 is the total number of reads in the samples. The asymptotic null distribution of 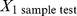 is a Chi-square with degrees of freedom equal to the rank of the matrix 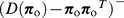, from which the statistical significance (P value) is calculated for the test.

#### Comparing 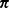 from two sample sets

Consider the problem of comparing microbiome samples between two groups of subjects (e.g., healthy versus diseased), or two body sites (e.g., oral versus skin). This can be done to test if two sets of microbiome samples are the same or different, such as is in a case-control study. This test is analogous to a two sample t-test in classical statistics, which, in our case, corresponds to evaluate whether the taxa frequencies observed in both groups of metagenomic samples, denoted by 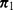 and 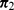, are equal.

The following statistic formally tests the hypothesis 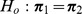 versus the alternative that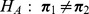 [32], [33]

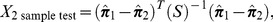
(7)which is a generalized Wald-type test statistics where 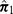 and 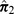 are the method of moments estimates, required for Wald-type statistics, of 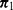 and 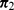, and 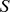 is a diagonal matrix given by
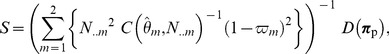
(8)where 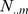 is the total number of reads in group m, 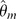 is the method of moments estimates of the overdispersion parameter of group m, 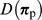 is a diagonal matrix with diagonal elements given by 
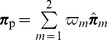
, a weighted average of estimated group means where 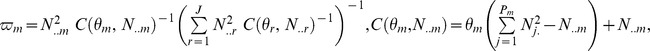 and 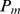 is the number of subjects in group m. The asymptotic null distribution of 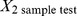 is Chi-square with degrees of freedom equal to 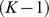, where 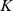 is the number of taxa, from which the statistical significance (P value) is calculated for the test.

#### Comparing 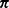 from more than two groups

Consider the problem of comparing microbiome populations between more than two groups of subjects (e.g., healthy, moderately sick, severely sick), or several body sites (e.g., saliva, subgingival and supragingival). This can be done to test if multiple sets of metagenomic samples are the same or different. This test is analogous to an analysis-of-variance test in classical statistics, which in our case corresponds to inquiry whether the taxa frequencies observed in multiple groups of microbiome samples, denoted by 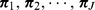, are equal.

The following statistic formally tests the hypothesis 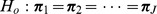 versus the alternative that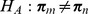 for at least one pair of groups [32], [33]

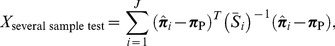
(9)which is a generalized Wald-type test statistics given by the weighted difference between each estimated group mean, 
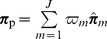
, a weighted average of the 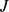 estimated group means, with weights 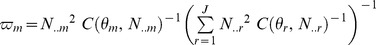, and 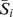 a diagonal matrix given by



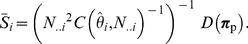
(10)The asymptotic null distribution of 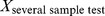 is Chi-square with degrees of freedom equal to 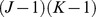, where *J* is the number of groups and *K* is the number of taxa, from which the statistical significance (P value) is calculated for the test. Note that there does not yet exist a multiple comparisons test analogous to Tukey’s Least Significance Difference or Duncan’s Range Test [34] routinely used in ANOVA to determine which groups are different when the omnibus rejects the null hypothesis, and is a focus of ongoing work in our lab.

### Power and Sample Size

When designing an experiment the goal is to simultaneously reduce the probability of deciding that the groups are different when they are not (Type I Error), and reduce the probability of deciding the groups are not different when in fact they are (Type II Error). From convention we often set the Type I Error = 0.05 (significance or P value) and the Type II Error = 0.2 resulting in power = 0.8, or 80% (power = 1– Type II error). The sample size needed to achieve these error rates depend on the probability model parameters, the hypothesis being tested, and the effect size indicating how different the groups are.

**Figure 2:**
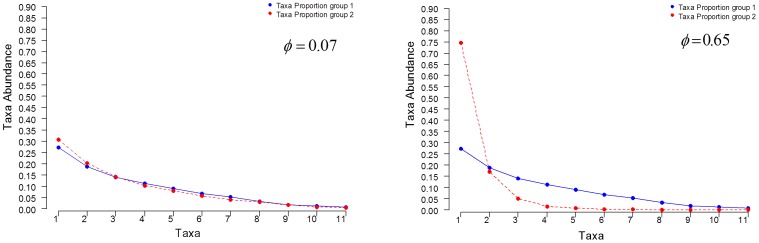
Definition of effect size. Illustration of a small and a large effect size when comparing two groups.

Power can be calculated in the R package for each of the four hypothesis tests discussed above, but for clarity we will only discuss comparison of 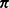 across two groups. Assume that the model parameters 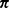 and 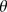 are known for each group, and we are interested in formally testing the hypothesis 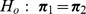 versus the alternative that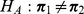. Intuitively, the effect size is defined by how far apart the vector of taxa frequencies 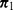 and 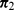 are from each other. There are several ways to quantify this. For example, a modified Cramer’s 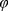 criterion can be used which ranges from 0, denoting the taxa frequencies are the same in both groups, to 1, denoting the taxa frequencies are maximally different (see Appendix S1 for more details). In Figure 2 we show examples of hypothetical data where the effect size is small (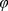 = 0.07) and large (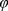 = 0.65) across two groups. It would be expected that more samples will be needed to test the 2 group comparison hypotheses for the small effect size than it would be for the large effect size parameters.

Power and sample size calculations are part of the R HMP package for the hypotheses presented in this paper [18]. The technical details of the mathematics for doing this are beyond the scope of this paper. We therefore have included for interested readers the mathematics for power and sample estimation in the Technical Report available from the authors.

### Performance Properties of these Tests

Statistical methods need to be tested for their performance to ensure the Type I and II error, P values, power and sample size calculations, and other results from their application are correct. This can be done analytically and proven mathematically, as well as through comprehensive Monte Carlo simulation studies. We chose the latter approach to confirm that these statistics behave as expected and present the results in the Technical Report available from the authors. We elected not to include these results in detail in this paper since it would detract from the primary goal of presenting statistical methods for applied analysis of metagenomic data. However, we briefly discuss those results which showed uniformly that these methods and software are valid.

We simulated Dirichlet-multinomial data for a variety of sample sizes, number of taxa, overdispersion, and effect size, and ran hypothesis tests for one sample, two sample and multiple sample comparisons. These simulations showed the Type I and II Error rates were as expected.

We performed simulated power and sample size calculations and obtained the correct results and show, as expected, the effect size, overdispersion, and sample size influence power. As the effect size increases, overdispersion decreases, or sample size increases, the power goes up. Of particular interest is that in some examples the number of reads also impacts power, with power increasing as the number of reads increases, holding effect size, overdispersion, and sample size constant. This appears to be related to the value of the overdispersion parameter, where for smaller overdispersion the number of reads has the greatest impact on power. Recall that as overdispersion goes to 0, the data converge to a multinomial distribution where the number of reads is known to have significant impact on power.

The Technical Report also presents several other tests of hypothesis that we did not include here since they seem less likely relevant to researchers. This includes comparing the overdispersion parameter across groups, and comparing distributions defined simultaneously by both 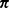 and 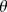.

### Results of Taxa Composition Data Analysis

In this section, we present results of analyses of metagenomic data from the 24 samples described above for saliva, subgingival and supragingival plaques analyzing the data at the class level. In our experience with metagenomic data analysis two types of analyses are routinely done. When the investigator is interested in community composition (what bacteria are there) the analysis proceeds with taxa labels preserved. In ecology this is usually known as analysis of species composition data [29], and here we will refer to this as taxa-composition data analysis. Alternatively, when the investigator is interested in community structure (what are the high level descriptions of the samples such as richness and diversity) the analysis proceeds without the taxa labels. In ecology this is called as analysis of rank abundance distribution (RAD) data [26]–[28]. The methods presented in this paper can be applied to both of these situations as illustrated below. In this section the samples are analyzed using a taxa-composition data analysis approach, and in the following section the same analyses are applied using a RAD data analysis approach. It should be noted that for these examples, when the taxa labels are ignored there is a loss of information in the data and the subsequent test of hypotheses show a decrease in power.

**Figure 3:**
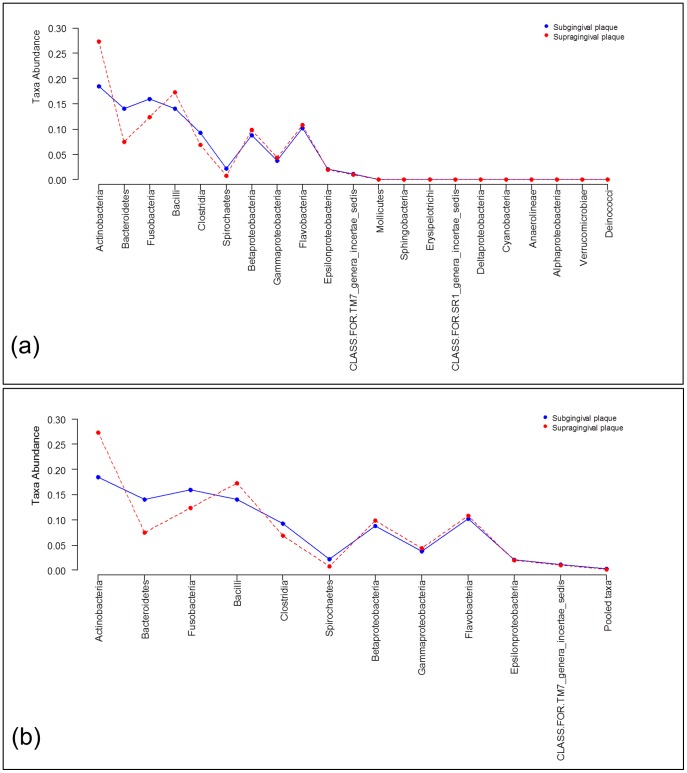
Comparison of two metagenomic groups using a taxa composition data analysis approach. Taxa frequency means at Class level obtained from subgingival plaque samples (blue curve) and from supragingival plaques samples (red curve): a) The mean of all taxa frequencies found in each group, b) The mean of taxa frequencies whose weighted average across both groups is larger than 1%. The remaining taxa are pooled into an additional taxon labeled as ‘Pooled taxa’.

One technical issue for the applied data analysis involves the presence of rare taxa. The test statistics proposed are based on the Chi-square distribution and the calculation of the P value is more precise when there are not many rare taxa. This is related to the technical issue of the convergence rate of the test statistic onto its Chi-square distribution. To improve the convergence rates of these test statistics all taxa frequencies whose weighted average across all groups is smaller than 1% are combined into a single taxon labeled as ‘Pooled taxa’. An illustration of the taxa composition data to be analyzed is shown in Figure 3 a) where we see that taxa from Mollicutes to Deinococci have low prevalence and found that their weighted average across both groups was less than 1%. In Figure 3 b) the same data are shown where these rare taxa are pooled, which are the data analyzed in the rest of this section. An alternative approach would be to drop the rare taxa.

### Multinomial versus Dirichlet-multinomial Test

Since overdispersion increases the Type 1 Error it is important to test if overdispersion is present in a set of microbiome samples. To do this we use Equation 5 to formally test the null hypothesis 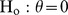 (implying no overdispersion) versus the alternative hypothesis 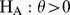 (implying overdispersion is present). In both subgingival and supragingival plaque samples, the null hypothesis that the data come from a multinomial distribution was rejected in favor of the Dirichlet-multinomial alternative. The overdispersion parameters, using method of moments (see Equation 2), are estimated to be greater than 0 and equal 0.047 for subgingival (T = 18,968; df = 11; P<0.00001), and 0.054 for supragingival (T = 18,953; df = 11; P<0.00001).

### Comparing 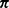 from Two Sample Sets

Consider the problem of comparing microbiome samples between the subgingival and supragingival samples to test if two sets of microbiome samples are different, such as is done in a case-control study. The application of Equation 7 hypothesis test to compare taxa frequencies (see Figure 3 b) 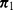 versus 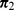 corresponding to subgingiva and supragingiva is significant (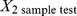 = 25.64; df = 11; P = 0.007). From this it is concluded that the null hypothesis that both taxa frequencies are the same is rejected in favor of the alternative that they are different.

### Power and Sample Size Calculation

Table 2 shows a power analysis to compare the taxa frequencies of the subgingival plaque versus the supragingival plaque populations from Figure 3b (effect size 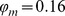) using 1% and 5% significance levels. To calculate power requires the Dirichlet-multinomial parameters, significance level, and specified number of subjects and reads to be defined. In this example the Dirichlet-multinomial parameters are obtained from the subgingival and supragingival 24 sample dataset, the significance levels based on conventional P-values, and a range of subject numbers and reads that could reasonably be obtained in the typical experimental setting.

**Table 2:** Power calculation as a function of number of sequence reads and sample size for the comparison of 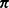 from the subgingiva and supragingiva populations, using as a reference the taxa frequencies obtained from the 24 samples, and 1% and 5% significant levels.

Alpha = 1%	Reads															
Subjects	500		1,000		2,500		5,000		10,000		20,000		50,000		1,000,000	
10	28.67%		29.45%		29.46%		29.83%		29.89%		30.00%		29.80%		29.95%	
15	54.25%		55.26%		55.50%		56.16%		56.16%		56.12%		56.57%		56.53%	
25	88.48%		89.44%		89.76%		90.03%		90.00%		90.11%		90.06%		90.04%	
50	99.95%		99.96%		99.97%		99.98%		99.96%		99.97%		99.97%		99.97%	
**Alpha = 5%**		**Reads**														
**Subjects**		**500**		**1,000**		**2,500**		**5,000**		**10,000**		**20,000**		**50,000**		**1,000,000**
10		51.96%		52.79%		53.14%		52.91%		53.20%		53.57%		53.16%		53.34%
15		76.01%		77.10%		77.90%		77.88%		77.98%		78.00%		77.92%		78.09%
25		96.50%		96.80%		97.02%		97.02%		97.13%		97.17%		97.09%		97.10%
50		99.99%		99.99%		99.99%		99.99%		99.99%		99.99%		99.99%		99.99%

Table 2 entries are the power achieved for the specified significance level, number of subjects, and number of reads. For example, for significance level = 1%, number of subjects = 15, and number of reads per subject = 10,000, the study has 56% power to detect the effect size observed in the data.

Note that the power is not impacted by increasing the number of reads. In this paper we show the results out to 1,000,000 expected reads per sample, but have conducted experiments running the number of reads out to 10,000,000 and reached the same conclusion. The likely cause of this is that increasing the number of reads does not impact the standard error around 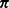, while increasing the number of subjects does. However, in experiments based on unlabeled taxa (i.e., rank abundance distributions) the number of reads does impact power.

### Comparing 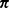 from Three Sample Sets

**Figure 4:**
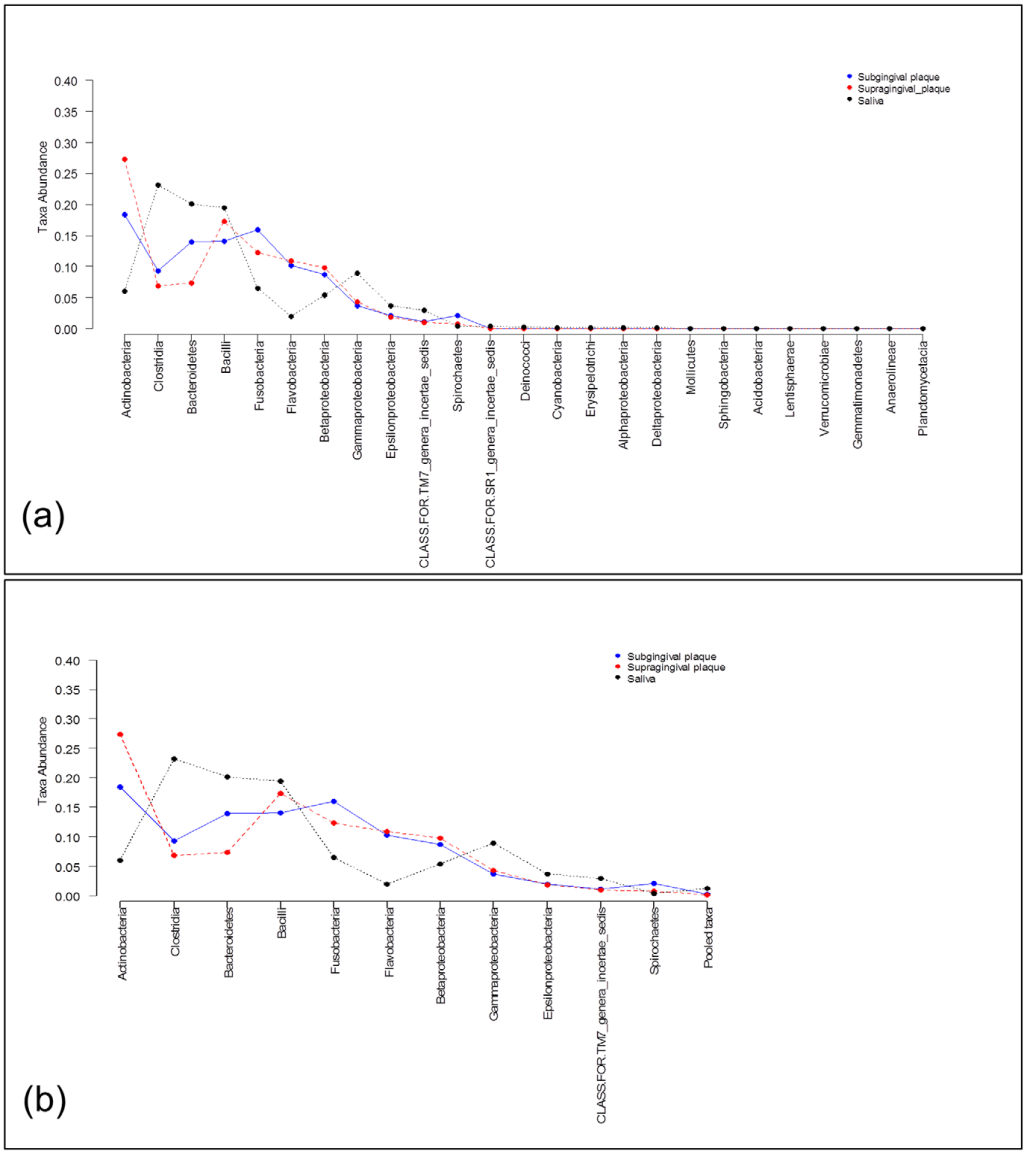
Comparison of three metagenomic groups using a taxa composition data analysis approach. Taxa frequencies at class level obtained from saliva (black line), subgingival plaque (blue line), and from supragingival plaques samples (red line): a) The mean of all taxa frequencies found in each group, b) the mean of taxa frequencies whose weighted average across both groups is larger than 1%. The remaining taxa are pooled into an additional taxon labeled as ‘Pooled taxa’.

It may be of interest to an investigator to compare three or more groups. Here, for purpose of illustration, we compare the saliva, subgingival and supragingival plaque populations from our 24 subjects. Figure 4 a) shows the taxa frequency to be analyzed where we see that taxa including Deinococci up to Planctomycetacia have very low prevalence. Following the same rationale as for the two sample comparison above, rare taxa were pooled, and the data analyzed is presented in Figure 4 b). It can be seen that the taxa here are the same as used in the comparison of subgingival versus supragingival plaque samples alone. To test if the saliva samples also are better fit to a Dirichlet-multinomial versus multinomial distribution we tested the hypothesis 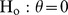 versus 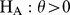 and conclude that in fact the Dirichlet-multinomial is the better distribution (P<0.00001).

The application of Equation 9 hypothesis test to compare taxa frequencies (see Figure 4) 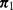 versus 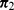 versus 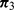 corresponding to subgingiva, supragingiva, and saliva is significant (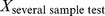 = 258.158; df = 22; P<0.00001). From this it is concluded that the null hypothesis that taxa frequencies across the three groups are the same is rejected in favor of the alternative that they are different.

The next step in this approach to hypothesis testing is to determine which of the groups are different. In the analysis-of-variance literature this is known as multiple comparisons. A simple approach calculates all pairwise P values and adjusts for the number of tests using a Bonferroni adjustment. In Table 3, we show the p-values (unadjusted and adjusted using Bonferroni) for all pairwise comparisons between saliva, supragingiva and subgingiva samples. This suggests that all three sample sets are statistically different.

**Table 3:** Unadjusted and Bonferroni adjusted p-values for all pairwise comparisons between saliva, supragingiva and subgingiva samples.

	Supraginigiva	Subgingiva
**Saliva**	P<0.00001 (unadjusted)	P<0.00001 (unadjusted)
	P<0.00003 (Bonferroni)	P<0.00003 (Bonferroni)
**Supragingiva**		P = 0.0007 (unadjusted)
		P = 0.0021 (Bonferroni)

### Result of Rank Abundance Distributions Data Analysis

**Figure 5:**
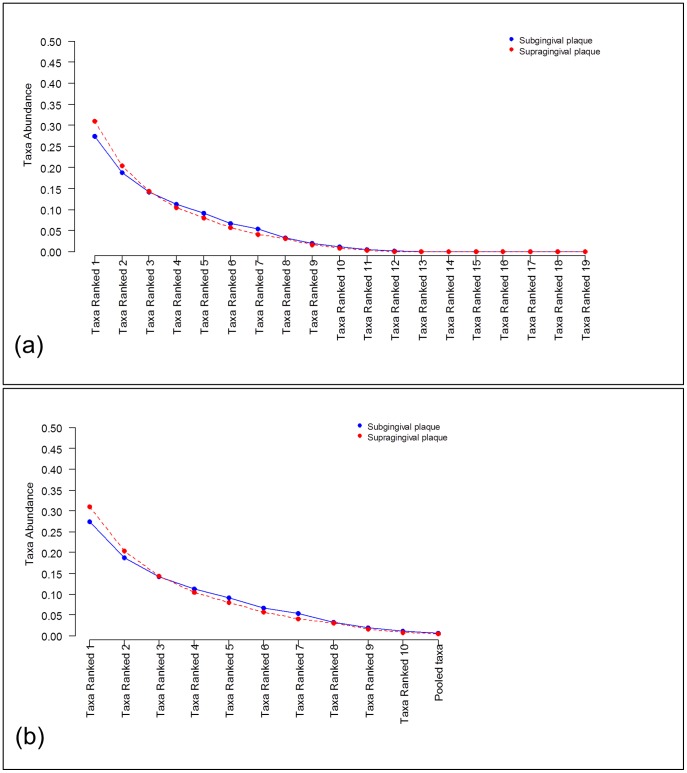
Comparison of two metagenomic groups using rank abundance distribution data. Ranked taxa frequencies mean at class level obtained from subgingival plaque samples (blue curve) and from supragingival plaques samples (red curve): a) The means of all ranked taxa frequencies found in each group; b) The mean of ranked taxa frequencies whose weighted average across both groups is larger than 1%. The remaining taxa are pooled into an additional taxon labeled as ‘Pooled taxa’.

Here we present the same analyses as in the previous example except using rank abundance distributions (RAD) which is of interest when the focus is on community structure (e.g., richness and diversity). Many analysts reduce each sample to a single measure of richness or diversity and then compare these values across groups. However, this results in a significant loss of information which should be avoided when analyzing data. The analyses presented here preserve most of the information (except taxa labels) which should prove to be more valuable for many situations. To illustrate, the RAD data to be analyzed in the following is shown in Figure 5 a) where we see that ranked taxa from 11^th^ to 19^th^ have low prevalence. In Figure 5 b) the same data is shown where these rare ranked taxa are pooled, which are the data analyzed in the rest of this section.

### Multinomial versus Dirichlet-multinomial Test

In both subgingival and supragingival plaque samples, the null hypothesis that the data come from a multinomial distribution was rejected in favor of the Dirichlet-multinomial alternative. The overdispersion parameters, using method of moments (Equation 2), are estimated to be greater than 0 and equal 0.008 for subgingival (T normalized = 69945; df = 215; P<0.00001), and 0.02 for supragingival (T normalized = 141301; df = 216; P<0.00001). Note that this hypothesis test establishes that the data are better represented by a Dirichlet-multinomial than a multinomial.

### Comparing 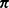 from Two Sample Sets

The application of the hypothesis test to compare ranked taxa frequencies (see Figure 5 b) 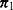 versus 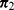 corresponding to subgingiva and supragingiva is not significant (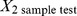 = 11.08; df = 10; P = 0.29). From this it is concluded that there is not enough evidence to reject the null hypothesis that ranked taxa frequencies are the same.

### Power and Sample Size Calculation

Table 4 shows a power analysis to compare the taxa frequencies of the subgingival plaque versus the supragingival plaque populations from Figure 5 b) (effect size 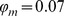) using 1% and 5% significant levels, respectively. To calculate power requires the DM parameters, significance level, and specified number of subjects and reads be defined. In this example the Dirichlet-multinomial parameters are obtained from the subgingival and supragingival 24 sample dataset, the significance levels set based on conventional P-values, and a range of subject number and reads that could reasonably be obtained in the typical experimental setting. The table entries are the power achieved for the specified significance level, number of subjects, and number of reads. For example, for significance level = 5%, number of subjects = 15, and number of reads = 10,000, the study has 40% power to detect the effect size observed in the data. Note that compared to the power calculations for the taxa composition data analysis (Table 2) the power is lower for the RAD comparison due to the smaller effect size observed in the data with this analysis.

**Table 4:** Power calculation as a function of number of sequence reads and sample size for the comparison of ranked 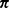 from the subgingiva and supragingiva populations, using as a reference the taxa frequencies obtained from the 24 samples, and 1% and 5% significant levels.

Alpha = 1%	Reads							
Subjects	500	1000	2500	5000	10000	20000	50000	1000000
10	8.57%	9.56%	10.06%	10.98%	10.51%	10.50%	10.62%	10.17%
15	15.88%	17.42%	18.91%	19.55%	19.85%	19.29%	19.32%	20.10%
25	36.36%	38.81%	41.65%	41.65%	42.91%	42.93%	42.66%	43.54%
50	81.81%	85.60%	87.38%	88.16%	87.50%	87.98%	88.30%	88.59%

Alpha = 5%	Reads							
Subjects	500	1000	2500	5000	10000	20000	50000	1000000
10	23.60%	24.60%	26.30%	22.80%	24.50%	28.20%	25.50%	25.70%
15	32.90%	38.70%	38.60%	40.10%	40.00%	39.10%	37.90%	43.00%
25	61.40%	63.50%	63.90%	65.60%	66.40%	64.90%	66.90%	67.10%
50	93.20%	94.80%	96.50%	95.30%	96.50%	95.40%	96.60%	97.40%

### Comparing 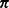 from Three Sample Sets

**Figure 6:**
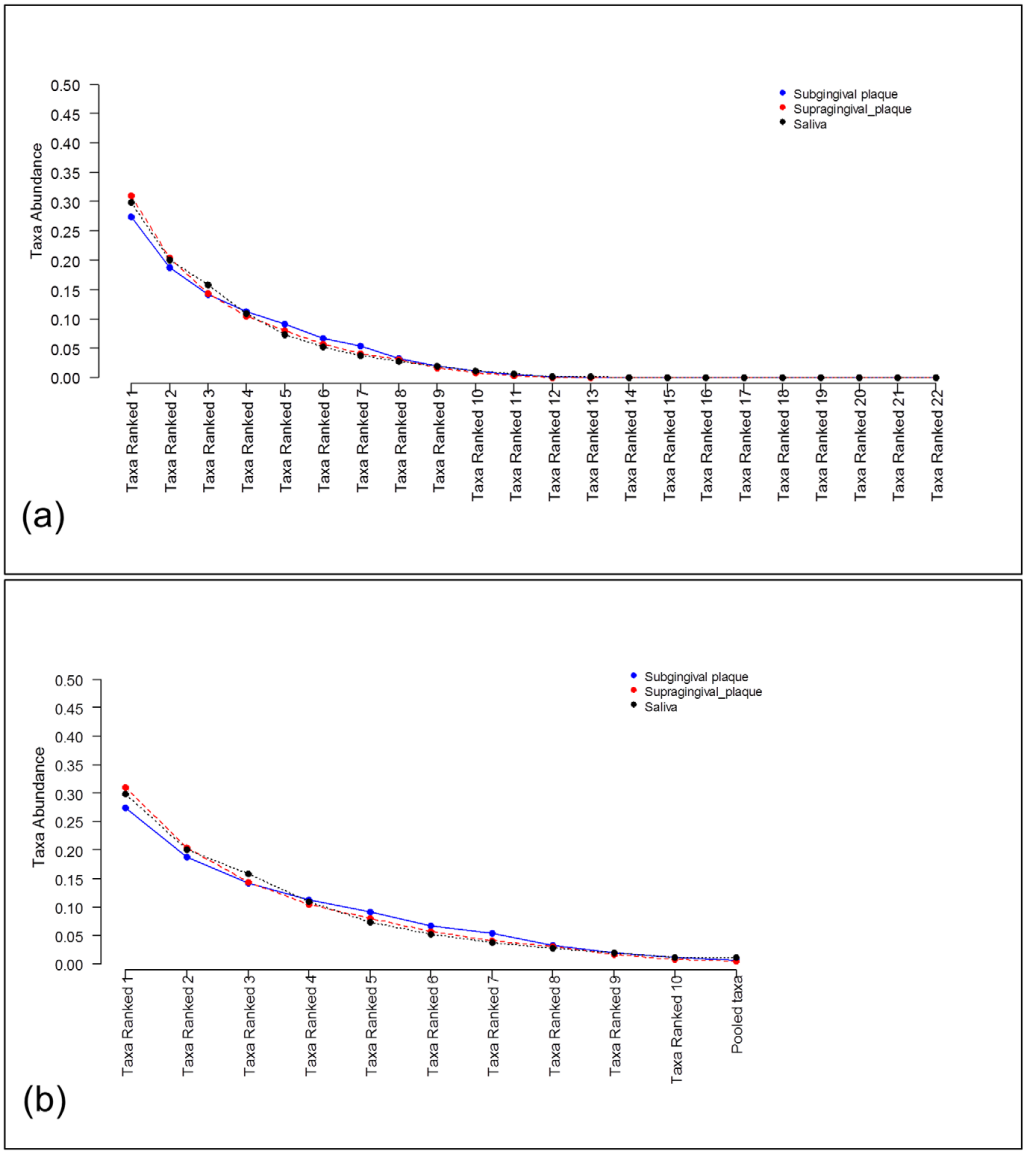
Comparison of three metagenomic groups using rank abundance distribution data. Ranked taxa frequencies mean at class level obtained from subgingival plaque samples (blue curve) and from supragingival plaques samples (red curve): a) The means of all ranked taxa frequencies found in each group; b) The mean of ranked taxa frequencies whose weighted average across both groups is larger than 1%. The remaining taxa are pooled into an additional taxon labeled as ‘Pooled taxa’.

Figure 6 a) shows the ranked taxa frequency to be analyzed where we see that ranked taxa between the 11^th^ to the 22^nd^ most abundant taxa have very low prevalence. Following the same rationale as for the two sample comparison above, ranked rare taxa were pooled, and the data analyzed is presented in Figure 6 b). It can be seen that the taxa here are the same as used in the comparison of subgingival vs supragingival plaque samples alone. To test if the saliva samples also are better fit to a Dirichlet-multinomial versus multinomial distribution we tested the hypothesis 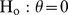 versus 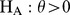 and conclude that in fact the Dirichlet-multinomial is the better distribution (P<0.00001).

The application of Equation 9 hypothesis test to compare taxa frequencies (see Figure 6 b)) 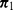 versus 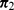 versus 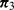 corresponding to subgingiva, supragingiva, and saliva is not significant (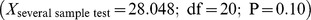. From this we concluded that there is not enough evidence to reject the null hypothesis that ranked taxa frequencies across the three groups are the same. Since the test of the three groups does not reject the null hypothesis the multiple comparison tests is not applicable.

## Discussion

The major contribution of this work is to begin formulating a biostatistical foundation for the analysis of metagenomic data. The Dirichlet-multinomial model is designed for count data and accounts for over dispersion, which if not adjusted for will result in increased Type I Error. The model gives rise to a broad class of statistical methods, including one sample and multi-sample tests of hypothesis, as well as calculating sample size and power estimates for experimental design. It also provides a set of parameters that can be interpreted analogous to the mean and variance of the bacterial diversity in a population. Computationally this model can accommodate large datasets consisting of multiple samples and essentially unlimited number of reads. For illustration of these methods we presented results of analyses and sample size/power calculations for three body sites for normal healthy individuals collected through the Human Microbiome Project.

Several issues that were referred to in the paper are discussed here. First, the performance of statistical tests depends on their behaving as predicted by statistical theory. For example, a test statistic under the null hypothesis should result in 5% of the tests being significant at the P< = 0.05 level. This and other measures of statistical performance have been confirmed through extensive simulation studies and are in a Technical Report available from the authors.

Second, the Dirichlet-multinomial model can be applied to taxa labeled and unlabeled data corresponding to Taxa composition and Rank Abundance Distribution (RAD) data analyses. In ecology this represents two alternative strategies focused on comparing individual species or diversity (RAD) across communities. The tools proposed here have general use in ecology, but we focused only on metagenomics in this paper. We leave it for others with in- depth experience in ecology to explain how these analyses can best be used in that field [26]–[29].

Third, in statistics a parametric model is usually preferred over a non-parametric models (e.g., permutation, bootstrapping) when available. In almost all cases parametric models are more efficient and require less data to achieve a given level of power. They also retain more information contained in the data (see the Introduction Section for a detailed discussion). Also, unlike non-parametric methods, our test statistics are appropriate when comparing groups that do not have the same within group variability, a common occurrence in microbiome data.

One of the potential limitations of our method is the incorporation of the rare taxa in the analysis. The performance of the test statistics proposed depends on their convergence to the Chi-square distribution which requires that on having rare taxa with a minimum frequency across subjects. Though, the proposed approached of ‘pooling rare taxa’ can be seen as loss of information, it currently stands as a practical approach which avoids giving importance to artificial rare taxa due to the effect of noise in the data. The analysis of rare taxa in metagenomic data is an ongoing topic of discussion and study; it is difficult to identify rare taxa from noise due to sequencing and classification errors, which is not the focus of these methods.

Several methods will be developed extending the Dirichlet-multinomial model for more complex metagenomic research designs and datasets. First, when parameters 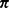 are shown to be different across groups, it is important to determine which taxa or ranked taxa are causing this difference. To avoid multiple testing problems from doing all univariate comparisons, methods analogous to linear contrasts from analysis-of-variance are being investigated. Second, application of the Dirichlet-multinomial to repeated measures, or mixed models analysis, can be used to monitor changes in the microbiome over time. Third, regression analysis adjusting for covariates can model changes in the microbiome such as how diet, age, or gender affects the stool microbiome. The three topics are current areas of research by the authors.

## Supporting Information

Appendix S1 **Measure of effect size.** **Introduction** **of a modified Cramer’s** ***φ*** **criterion such that it does not depend on the sample size when the test statistics takes into account the overdispersion.**
(DOCX)
