# Supplementary material for: Hypothesis Testing and Power Calculations for Taxonomic-Based Human Microbiome Data
Source: PLoS One. 2012 Dec 20;7(12):e52078. doi: 10.1371/journal.pone.0052078 (PMC3527355; doi:10.1371/journal.pone.0052078)
Supplement: Appendix S1 — Measure of effect size. Introduction of a modified Cramer’s φ criterion such that it does not depend on the sample size when the test statistics takes into account the overdispersion. (DOCX) [file pone.0052078.s001.docx]

A**ppendix S1: Measure of effect size**

The classical Cramer’s $\varphi$ criterion, is defined by

$\varphi=\sqrt[2]{\frac{\chi^{2}}{N (\min\left( J,K \right)-1)}}$,

where $\chi^{2}$ is a chi-square test statistics, namely a test statistics which follows a chi-square distribution (or asymptotically) when the null hypothesis is true,$N$ is the total sample size, $J$ is the number of groups being compared, and $K$ is the total number of taxa. However, for the test statistics presented in this work, the Cramer’s $\varphi$ criterion has the undesired property that it depends on the sample size and number of reads. Therefore, we propose a modified version of Cramer’s$\varphi$, denoted here by $\varphi_{m}$, such that its dependency on the sample size and number of sequence reads is nullify,

$\varphi_{m}=\sqrt[2]{\frac{\chi^{2}}{\chi_{max}^{2}}}$,

where $\chi_{max}^{2}$ is the value of the Chi-square statistics for the maximum difference between the taxa frequency means being compared. For example in the case of a two sample mean comparison the maximum difference between the mean of the taxa frequency is achieved when every group has one taxa each which are non-overlapping across groups. Note that for the one-sample and two-sample mean test comparisons it is straightforward to show that when there is not overdispersion$\chi_{max}^{2}=N$, and since$\min\left( J,K \right)=2$, then $\varphi_{m}$ is equal to the Cramer’s $\varphi$ criterion.
